# HOPE: Help fOr People with money, employment, benefit or housing problems: study protocol for a randomised controlled trial

**DOI:** 10.1186/s40814-017-0179-y

**Published:** 2017-09-19

**Authors:** M. C. Barnes, A. M. Haase, A. M. Bard, J. L. Donovan, R. Davies, S. Dursley, J. Potokar, N. Kapur, K. Hawton, R. C. O’Connor, W. Hollingworth, C. Metcalfe, D. Gunnell

**Affiliations:** 1 School of Social and Community Medicine, University of Bristol, Bristol, BS8 2PS UK; 2 National Institute for Health Research Collaboration for Leadership in Applied Health Reserach and Care West, UH Bristol NHS Trust, Bristol, UK; 3 School of Policy Studies, University of Bristol, Bristol, UK; 4 School of Veterinary Sciences, University of Bristol, Bristol, UK; 5 Public Patient Involvement, University of the West of England, Bristol, UK; 6 Psychiatric Liaison Team, UH Bristol NHS Trust, Bristol, UK; 7 Centre for Suicide Prevention, University of Manchester, Manchester, UK; 8 Centre for Suicide Research, University of Oxford, Oxford, UK; 9 Suicidal Behaviour Research Laboratory, University of Glasgow, Glasgow, UK; 10 NIHR Biomedical Research Centre at the University Hospitals Bristol NHS Foundation Trust and the University of Bristol, Bristol, UK

**Keywords:** Psychosocial intervention, Self-harm, Financial difficulties, Pilot study, Motivational interview methods

## Abstract

**Background:**

Self-harm and suicide increase in times of economic recession. Factors including job loss, austerity measures, financial difficulties and house repossession contribute to the risk. Vulnerable individuals commonly experience difficulties in navigating the benefits system and in accessing the available sources of welfare and debt advice, and this contributes to their distress. Our aim is to determine the feasibility and acceptability of a brief psychosocial intervention (the “HOPE” service) for people presenting to hospital emergency departments (ED) following self-harm or in acute distress because of financial, employment, or welfare (benefit) difficulties.

**Method:**

A pilot study including randomisation will be employed to determine whether it is possible to undertake a full-scale trial. Twenty people presenting to the ED who have self-harmed, have suicidal thoughts and depression and/or are in crisis and where financial, employment or benefit problems are cited as contributory factors will be asked to consent to random allocation to the intervention or control arm on a 2:1 basis. People who require secondary mental health follow-up will be excluded. Those randomised to the intervention arm will receive up to six sessions with a mental health worker who will provide practical help with financial and other problems. The mental health worker will use the motivational interviewing method in their interactions with participants. Control participants will receive one session signposting them to existing relevant support organisations. Participants will be followed up after 3 months. Participants and the mental health workers will take part in qualitative interviews to enable refinement of the intervention. The acceptability of outcome measures including the PHQ-9, GAD-7, repeat self-harm, EQ5D-5L and questions about debt, employment and welfare benefits will be explored.

**Discussion:**

This study will assess whether a full-scale randomised trial of this novel intervention to prevent self-harm among those distressed because of financial difficulties is feasible, including the acceptability of randomisation, potential rate of recruitment and the acceptability of outcome measures.

**Trial registration:**

ISRCTN58531248

**Electronic supplementary material:**

The online version of this article (10.1186/s40814-017-0179-y) contains supplementary material, which is available to authorized users.

## Background

Economic recessions are usually accompanied by increases in unemployment and economic hardship [1]. These changes are associated with rises in suicide and self-harm [2–6]. People with pre-existing mental health problems are the most vulnerable [7, 8]. However, even in times of prosperity, job loss and debt are associated with depression and suicide risk [9, 10].

The literature has tended to focus on the barriers to accessing services for welfare, debt and employment advice (e.g. [11, 12]). The few randomised controlled trials (RCTs) of interventions to offset the impact of financial, employment or welfare benefit difficulties are mostly focussed on those who have lost their job (see, e.g. [13, 14]) rather than individuals experiencing financial pressures for a range of reasons. To the authors’ knowledge, there have been no RCTs specifically targeting those who have self-harmed or are in distress where financial, employment, welfare benefit or housing difficulties are a contributory factor.

Our recent qualitative research has highlighted that vulnerable individuals commonly experience difficulties in finding their way through the benefits system and in accessing available sources of welfare and debt advice [8, 15]. This is particularly the case for people with pre-existing mental health problems and whose self-harm was precipitated by financial, employment, benefits or housing problems arising from financial difficulties [8].

Informed by the findings from our research [3, 4, 8, 15], we have developed the “HOPE (Help fOr People with money, Employment, benefit or housing problems) service” for people who have self-harmed and/or presented to the emergency department (ED) in psychological distress and where financial, employment, benefits or housing problems were contributory factors. The intention is that the intervention will support participants through the period of acute distress to the resolution of their problems. We also intend that the intervention will enable the participant to feel more confident in dealing with similar difficulties in the future.

### Aims of the study

Before proceeding to an RCT of a complex intervention, it is important to ensure that the intervention and delivery is fully developed, feasible and acceptable [16]. An exploratory pilot trial can also demonstrate the feasibility of evaluating the intervention in a trial and the randomisation and recruitment processes in particular. Our overall aim, therefore, is to determine the feasibility and acceptability of the HOPE service itself and its evaluation in a pilot RCT before undertaking a full pragmatic RCT. Specific objectives are to gain initial estimates of the recruitment rate that can be achieved (indicating the acceptability of evaluation in a randomised trial); of adherence to the intervention (indicating the acceptability of the intervention); and of the completeness and appropriateness of outcome measurements [17]. This pilot study employs primarily qualitative research and process evaluation [18] techniques to understand the feasibility aspects.

### Objectives

The objectives of this study are as follows:

1. Explore the acceptability of randomisation and agreement to accept the allocation to intervention or control arm
2. Explore the acceptability of the content of the intervention and control arms to participants and staff
3. Estimate likely recruitment rates to a full trial and identify opportunities to increase recruitment
4. Identify recruitment pathways and optimise these
5. Estimate likely loss to follow-up
6. Identify additional training needs of the service providers
7. Explore the acceptability of outcome measures (health and economic)

## Methods

### Intervention

The intervention was developed over the period of a year in several stages. Results from phase I of the study, which consisted of in-depth interviews [8, 15], quantitative analyses [3, 4] and literature reviews [19, 20], were used to guide a workshop involving a range of stakeholders including service user research advisors, representatives from debt advice organisations, Samaritans (a suicide prevention charity), the UK Government’s Department for Work and Pensions (DWP) and academics working in public health, primary care, psychiatry, sociology and clinical trials. The main recommendations from the workshop were (1) to prepare a policy document [21] describing the research findings and (2) to develop a practical intervention that would be generalisable across England, to help people experiencing psychological distress in the context of financial, employment, housing or welfare benefit problems. The qualitative study had identified the need for such an intervention [8, 15] as many participants struggled to access and use available statutory and voluntary sector organisations for their economic problems, as a result of psychological distress.

In phase II, one of the authors (MB) made contact with several service provider organisations in Bristol (advice organisations, e.g. Citizens Advice Bureau/a free debt advice agency, hospital staff, social housing organisations, foodbanks) to better understand existing service provision. Following this, a navigator-style intervention was proposed: a role involving guiding service users to the available support organisations. The target population will be patients who attend hospital with either self-harm or in acute distress where financial, employment, housing and benefit-related difficulties are contributory. The intervention will consist of up to six 1-h sessions of practical help guiding participants through the complex system of voluntary and statutory service support organisations, building their confidence to eventually manage their own affairs again. The intervention will be delivered by a team of six individuals (“HOPE workers”) trained to use a range of motivational interviewing (MI) methods [22] (see Table 1). Staff will have a minimum of 2 years’ experience working with people with mental health needs and of carrying out needs and risk assessments and the support planning process. HOPE workers will be employees of a mental health charity which offers housing and support to people with different kinds of mental health problems across the west of England (www.second-step.co.uk).

**Table 1:** Details of HOPE intervention

The HOPE sessions are flexibly tailored to suit the participant and their progress.
The expected frequency is for participants to receive up to six 1-h sessions over a 2-month period.
The intervention will take place in the service user’s homes, the service providers’ office or place of the service user’s choosing.
The HOPE worker may travel with the participant to other organisations, e.g. debt advice agencies.
Tasks for the HOPE worker will include:
• Assessment of need and creating a support plan.
• Helping with correspondence/interpretation of DWP letters.
• Welfare benefits advice.
• Support in accessing key agencies (such as benefits or free debt advice).
• Supporting and connecting with other community resources, including mental health care.
• Participants are free to stop the sessions before they have received all six if they feel they are no longer useful.

### Theoretical rationale

Self-determination theory (SDT) [23] emerged in discussion as the most appropriate model allied to the aims of the intervention. SDT suggests that for an individual to modify their behaviour, core psychological needs must be met: specifically, the need for an individual to have control and choice over activity (autonomy) and to feel capable of doing something (competence). Furthermore, these needs can be met by providing an autonomy supportive environment created through the use of supportive communication. Motivational interviewing (MI) is a directive, client-centred communication style for helping people explore and resolve ambivalence, in order to move towards change [22]. The underlying principle of MI connects to self-determination theory, working with the service user to increase their independence, decision-making and confidence when approaching and dealing with their problems. Behaviour change techniques based on Michie’s taxonomy model [24] have also been reviewed for this population, although there is limited evidence to suggest how best to apply these techniques within a mental health setting. As such, focused application of MI methods and principles as a comprehensive communication model was selected in order to guide the HOPE workers through supporting individuals to work towards moving beyond ambivalence.

#### Training

HOPE workers will receive two half-day training courses delivered by an MI trainer and health psychologist, covering the underpinning principles of MI and SDT—the principal behaviour change strategies relevant to the intervention—and an introduction to communication using MI methods. A comprehensive manual will be provided describing the aims of the HOPE service, outlining the session structure and how to deliver the intervention. Training sessions will be evaluated by observation and feedback. HOPE workers will have access to monthly group supervision and 1:1 support by the health psychologists who developed the intervention (AH/AS) when needed throughout the trial.

### Control

Participants randomised to the control arm will receive a one-off session with the HOPE worker who will identify the greatest needs along with the service user, signpost them to appropriate agencies and leave relevant reading materials. The technical components (to do with strengthening the language of change) of the MI method will not be used in this interaction.

### Usual care

Participants in both arms will receive usual care in addition to the one-off or six sessions with the HOPE worker. Participants in both arms of the trial will have been discharged from hospital back to the care of their GP with no specialist follow-up; usual care will therefore consist of the care they would normally receive from their GP, social services and the voluntary sector.

### Patient and public involvement (PPI) in developing the research

A group of three service user advisers with lived experience of job loss, unemployment, financial problems, self-harm and mental health problems helped design the intervention and have commented on all recruitment, question and interview documents.

### Recruitment

#### Setting and sample

Potentially eligible patients aged 18 + years will be identified and recruited (first phase) by members of the liaison psychiatry team at an acute hospital serving a large inner city area of England. Potential eligibility will be assessed by a clinician on the basis of information provided in the psychosocial assessment in the ED. They will be patients who have self-harmed, had suicidal thoughts, have depression and/or were in crisis and where financial, employment, housing or benefit problems were cited as contributory factors. Full eligibility criteria are listed in Table 2.

**Table 2:** HOPE pilot eligibility criteria

Inclusion criteria	18 + years
	Men and women
	People who have self-harmed and/or are in psychological distress but do not meet the criteria for secondary mental health care referral of continuing help by support agencies
	People whose psychosocial assessment indicates that job loss, difficulties finding a job, benefit changes and/or sanctions (actual or fear of changes and sanctions), housing problems or debt and economic hardship as a result of financial problems were a contributory factor to their distress/self-harm
Exclusion criteria	People referred for secondary care specialist psychiatric community or inpatient services
	People with a support worker delivering similar or same support as HOPE workers
	People experiencing a psychotic episode, have thought-disorder or who are unable to give consent
	People with addiction as their primary problem
	People not fluent in English (due to insufficient funding for translation services)
	People living outside of the catchment area for the HOPE service

##### Phase I

Following psychosocial assessment, the clinician will screen the patient on the eligibility criteria, explain the study and intervention and provide an invitation letter and information sheet to eligible patients interested in participating (Additional file 1: PIS short). The patient will be asked for their consent to being contacted by the HOPE team and a researcher after leaving hospital (Additional file 2: PIS long). If the patient gives consent, their details are passed on to the researcher and HOPE service (Additional file 3: Consent to contact).

##### Phase II

A HOPE worker will make telephone contact with the potential participant within 3–5 days of their discharge from hospital. An appointment will be made for the researcher and HOPE worker to visit the participant at home—or wherever the participant prefers—within the following week of this contact. At this meeting, the study and service will be discussed with the potential participant, randomisation explained, and consent and baseline measures taken by the researcher (Additional file 4: Consent). If the potential participant agrees to randomisation, the researcher phones the allocation service and randomisation will take place in either arm of the study.

**Fig. 1:**
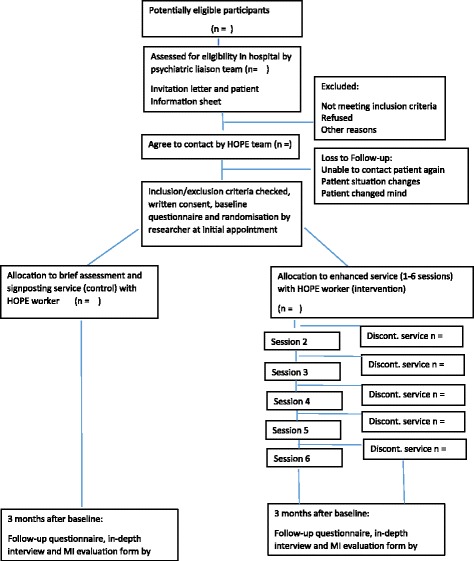
HOPE flow chart

For the purposes of this pilot, the first two participants are allocated to the intervention—to allow any necessary amendments to the service process from worker feedback and ensure skills learnt during the MI training are used as soon after training as possible—and the subsequent 18 allocated in a simple random order to intervention (*n* = 12) or control (*n* = 6). Following the completion of consent, baseline data collection and randomization, the researcher informs the participant of their treatment allocation and leaves; the HOPE worker will continue with either the brief assessment and signposting service (control arm) or the first session of up to six sessions of the enhanced service (intervention). The flow of assessment, recruitment and randomisation is shown in Fig. 1.

Through the use of audio-recording recruitment consultations between staff and patients and observation of everyday departmental procedures, it will be possible to identify where there may be clear as well as more hidden obstacles in the recruitment process. This can then be fed back to staff in a positive fashion to help optimise recruitment [25]. It is not necessary for participants to agree to audio recording of consultations to take part in the study. If they decline, the recruitment and randomisation process continues as usual.

A log will be kept of the number of people identified as eligible; the number approached for participation by the psychiatric liaison staff and the numbers who agree to be randomised. These are different categories. At the initial stage of the study, we suspect some liaison team members may forget to recruit eligible participants, and one role of the feasibility study is to identify approaches to minimise such lost opportunities.

To ensure allocation concealment, once a participant has agreed to take part and baseline measures are recorded, the researcher will telephone the study office, will log the participant into the study and will then be told the allocation. The reason for rejection of allocation will be recorded.

The intervention will be discontinued if the participant is proved ineligible due to receiving similar support through another service. Participants may often have other support agencies involved in their care which may or may not help them with, e.g. benefits entitlements or debt advice. We felt it was important to avoid duplication and possibly conflicting messages or overlap in the support offered to this vulnerable population, which would also make evaluation difficult. HOPE workers check throughout the sessions that the participants do not have similar support in place; if this occurs, the service is discontinued.

The HOPE team will maintain close contact with the participants, keeping appointments flexible and re-scheduling missed appointments to aid adherence to the intervention.

### Design

This is a pilot randomised controlled trial using block randomisation to ensure a 2:1 allocation ratio to the intervention or control. A 2:1 ratio was chosen for the pilot to allow more data to be gathered on the intervention; if a main study goes ahead, it is likely that a 1:1 allocation ratio will be used.

### Process evaluation

Quantitative and qualitative approaches will be used to evaluate the pilot HOPE service and research procedures. Baseline measures will be completed by participants at the time of recruitment (prior to randomisation) and outcome measures at 3 months following randomisation. In-depth qualitative interviews with the service users and the HOPE workers will explore their views of the service provided and research procedures.

A flexible, iterative process is necessary during pilot studies enabling any screening or recruitment difficulties to be identified and addressed to facilitate more effective recruitment [25]. During recruitment, MB will make weekly visits to the psychiatry liaison team offices at the hospital to monitor the recruitment pathway and rate and gather views on the acceptability of the intervention from staff. This is to understand recruitment as it happens and to develop a plan of action to address identified difficulties and to optimise informed consent. Recruiting patients with mental health problems has previously been identified as difficult for clinicians [26]. The ethics committee will be notified of any modifications to the protocol.

During the study, the HOPE workers will maintain records in which they document details of their interactions with service users, time logs, services contacted, appointments attended, MI techniques used and reflections on MI practice. Audio-recordings will also be taken (with service user consent) of some sessions to be used in the regular de-briefing and feedback sessions with HOPE workers and MI trainers to inform further training and manual development for the trial.

The MI experts/trainers will also keep a record of the training sessions, number of attendees, timings and content of sessions.

The HOPE workers will be interviewed about their views of the service and their experience of being trained and delivering the intervention. Alongside the interview data about the service from the service users, a short questionnaire for evaluating the motivational interviewing part of the intervention will also be completed by participants.

A sample size of 20 was felt to be sufficient to make any changes necessary to the intervention manual, test and modify procedures and outcome measures and estimate rates of recruitment and loss to follow-up.

### Outcome measures

As this is a pilot study, we do not have sufficient statistical power to detect differences in the quantitative measures we will record. Our qualitative interviews will guide us concerning appropriate outcomes for a full trial. Participants will complete a questionnaire including a validated measure of depression (the PHQ-9) [27], a validated measure of anxiety (GAD-7) [28], a validated general health questionnaire (the EQ-5D-5L) [29] and a financial self-efficacy scale (FSES) [30] at baseline and follow-up. Incident episodes of self-harm will be identified from the local self-harm register. We anticipate that PHQ-9 scores or self-harm are likely to be the primary outcome measures in a full trial. Our baseline and follow-up questionnaires will also record sociodemographic information and include a series of questions concerning employment, finances, debt and welfare benefits received drawn from the Do Well Study [31], the ONS [32] and the Census [33].

Three months after randomisation, the researcher will meet with the participant to complete the follow-up questionnaire with an extra short questionnaire to evaluate MI interaction [34] and an audiotaped, semi-structured qualitative interview will be conducted with the participant. Three months is considered to be long enough for six sessions to have been delivered, taking into account likely delays in making and maintaining contact in this patient population. We will attempt to follow up all participants regardless of whether they complete the intervention. Participants are asked to give their own details as well as those of a family member or friend in case it is difficult to contact them directly.

The interviews will explore participants’ perceptions and experiences of the HOPE service in relation to their self-harm or psychological distress that prompted ED attendance, their financial difficulties and whether it was acceptable as an intervention. Other topic guide areas will include barriers and facilitators to using the service and accessing subsequent services; participants’ current situation; their feelings and plans for the future in relation to their financial situation and mental health. Participants will also be asked about the health care use in the previous 3 months and if they have contacted any other support services for the financial worries. Finally, they will be asked about the process of recruitment to the research and randomisation.

Clearly, the researcher will not be blind to arm allocation at the follow-up interview. Given the nature of the feasibility study (including a detailed follow-up interview) and limited resources, this was unavoidable. The questionnaire is self-completed, which avoids the possibility of interviewer bias. In a full trial, we anticipate that the follow-up interview would be conducted by a researcher blind to intervention arm, but some disclosure is likely to occur during the course of the interview.

Procedures to safeguard patient well-being during the recruitment process describe steps to take if the interviewee appears distressed during the interview starting with the researcher offering support during the interview to contacting the study clinician. Previous findings suggest individuals are more likely to derive benefit from participation in qualitative research than experience harm [35].

Table 3 shows the time points at which data and outcomes are measured.

**Table 3:** Time points at which measures and data collected

	Recruitment	Allocation	During intervention	3 months
Eligibility screening	X			
Consent to contact	X			
Informed consent		X		
Baseline questionnaire^a^		X		
Intervention				
Control (1 session)				
- Case notes recorded			X	
Intervention (1–6 sessions)				
- Case notes recorded			X	
Outcomes				
Follow-up questionnaire^b^				X
Face-to-face interview				X

^a^Includes basic socio-demographic information, PHQ-9, GAD-7, EQ-5D-5L and questions concerning employment, finances, debt and welfare benefits received drawn from the Do-Well Study [30], the ONS, the Census and FSES (see the ‘Outcome measures’ section)

^b^As above plus adapted MIMSI questionnaire (motivational interviewing measure of staff interaction)

#### Qualitative data analysis

The audio-recorded interviews will be transcribed and systematically coded using NViVo software. Data will be analysed using case studies analysis [36] to include the context and various data sources for each participant, with particular emphasis on the similarities and differences within and between cases according to the number of sessions attended. A case studies comparison exercise will be used with members of the research team and an independent researcher to lead to consensus.

#### Quantitative analysis

Simple descriptive analyses of the proportion of eligible participants who (a) consent to contact; (b) are randomised and (c) are followed up at 3 months will be carried out. Similarly treatment adherence and completeness of responses will be presented as a CONSORT flow chart. Questionnaire responses will be presented as simple summary statistics by allocated trial arm. Data will be analysed according to the randomisation arm (intention to treat analysis).

#### Data management

Data about potential or enrolled participants will be shared between the researcher and HOPE team on the nhs.net password-protected system. All electronic records will be kept by HOPE workers on password-protected office computers. Paper records will be kept in locked filing cabinets. Similarly, all data will be kept on password-protected university computers and locked filing cabinets on university property. Qualitative data will be collected on encrypted audio recorder, and transcription will be carried out by one university staff member who has signed a confidentiality agreement. Data used in reports will be anonymised. There is no planned public access to data aside from the trial investigators.

#### Dissemination

Results will be disseminated through conferences, peer reviewed articles and reports to funders. Participants have the option of receiving a summary of the results if agreed on the consent form. Recruitment, retention and summary statistics will be made available along with the CONSORT diagram on the National Institute for Health Research (NIHR) website.

### Steering committee/management information

The project will be overseen by a steering committee comprising academics experienced in trial design (AH, DG, JD, NK, KH, RO’C, WH, CM) and qualitative methodology (JD, MB), clinicians and academics experienced in mental health interventions (AH, JD, NK, KH, RO’C, CM, RD, JP, SD, DG), a service user (RD), a statistician (CM) and health economist (WH). Sub-groups of the stakeholders implementing the service/study and the research group will also meet on a regular basis. There is no data monitoring committee.

## Discussion

This pilot study aims to assess the feasibility of a full-scale RCT of a theory-based intervention as a means of supporting people who have self-harmed or presented to ED with distress through a period of crisis associated with financial, employment, housing or benefit troubles. We also aim to increase participants’ levels of confidence/self-efficacy in dealing with future financial difficulties in the longer term. The pilot is targeting anyone where the specified socio-economic difficulties contribute to their ED presentation, but their mental health difficulties are not judged by the psychiatric liaison team as severe enough to warrant referral to secondary mental health care. It may then be appropriate to evaluate the intervention in populations that have presented in other (non-hospital) settings and among participants who have not self-harmed or are yet to develop acute distress, for example, in populations where people may have defaulted on their rent to the local council, which can often be the first sign of financial difficulties. This decision to evaluate this will be made by the Steering Committee members based on evidence from parallel strands of research investigating appropriate alternative sources of recruitment (Citizens Advice Bureaux; Debt Advice Agencies etc.) and the feasibility of delivering this intervention to participants in the present trial.

The decision to proceed to a full pragmatic RCT will be made by the study Steering Committee. There are no independent members on the Steering Committee nor specific stop/go criteria. This is very much an exploratory feasibility study, and funding for the full trial has not been obtained. Initially, we will need to make judgements based on the feasibility data; these will be based on the rate of recruitment (and so the number of centres needed to be involved in recruitment); likely trial costs relative to the value of the research question; qualitative feedback from participants in receipt of the intervention; and loss to follow-up.

Given the modest scale of this pilot study, a full pragmatic RCT will include an internal pilot study to establish the recruitment rate at each participating centre, with the decision to continue to a full trial to completion being informed by pre-specified stop-go-amend criteria.

### Pilot status

Recruitment to the pilot trial concluded in February 2017.

## Additional files

Additional file 1: Patient information sheet (short). (DOCX 35 kb)

Additional file 2: Patient information sheet (long). (DOCX 70 kb)

Additional file 3: Consent to contact. (DOCX 77 kb)

Additional file 4: Consent. (DOCX 76 kb)

Additional file 5: SPIRIT checklist. (DOC 121 kb)
