# Supplementary material for: HOPE: Help fOr People with money, employment, benefit or housing problems: study protocol for a randomised controlled trial
Source: Pilot Feasibility Stud. 2017 Sep 19;3:44. doi: 10.1186/s40814-017-0179-y (PMC5629806; doi:10.1186/s40814-017-0179-y)
Supplement: Supplementary file 1 — Patient information sheet (short). (DOCX 35 kb) [file 40814_2017_179_MOESM1_ESM.docx]

HOPE:

Help for people with money, Employment or benefit problems


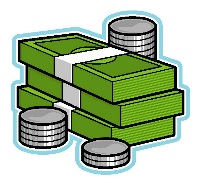
Are you struggling or experiencing distress due to financial hardship, debt, job loss or benefits worries?

Would you like to see if extra help to get advice and support about these matters might make a difference and help you to be more confident about dealing with financial difficulties in the future?

The Liaison Psychiatry services at the BRI are currently helping with a study called HOPE that is being run by researchers at Bristol University. At the moment people experiencing distress due to financial hardship, debt, job loss or benefits worries are usually given leaflets about support organisations when they leave hospital. The study has been set up to see whether being given extra help to get relevant advice and support on these matters makes a difference to peoples’ wellbeing and financial situation.

You have been given this leaflet because the person from the Psychiatry liaison team you have seen thinks you may benefit from this service. We would like to tell you briefly about the study and see if it is something you would like have more information about.

The study is comparing two ways of offering this support but both include being offered the support of a HOPE worker who will talk with you about your situation and financial problems. Together you will decide on the best action to take and the HOPE worker will help you to get the support you need.

People who take part will receive either:

Meeting with the HOPE worker for one session of an hour and a half OR


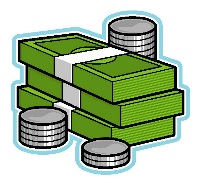
Meeting with the HOPE worker for up to 6 sessions of one hour

Allocation to the two different ways of giving the service is decided by chance: a process called randomisation. This means that you will have an equal chance of having either one session or six.

To assess which service is most effective, people taking part will be asked to complete questionnaires and take part in an interview about their experience of the HOPE worker service.

**At this time, we are only asking if you might be interested. If you would like to have more information, a member of the liaison team will ask for your permission to be contacted by one of the research team, who can give you more information.**

**Thank you for reading this leaflet**
