# Supplementary material for: HOPE: Help fOr People with money, employment, benefit or housing problems: study protocol for a randomised controlled trial
Source: Pilot Feasibility Stud. 2017 Sep 19;3:44. doi: 10.1186/s40814-017-0179-y (PMC5629806; doi:10.1186/s40814-017-0179-y)
Supplement: Supplementary file 2 — Patient information sheet (long). (DOCX 70 kb) [file 40814_2017_179_MOESM2_ESM.docx]

| **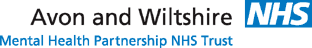** | 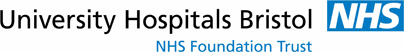 | 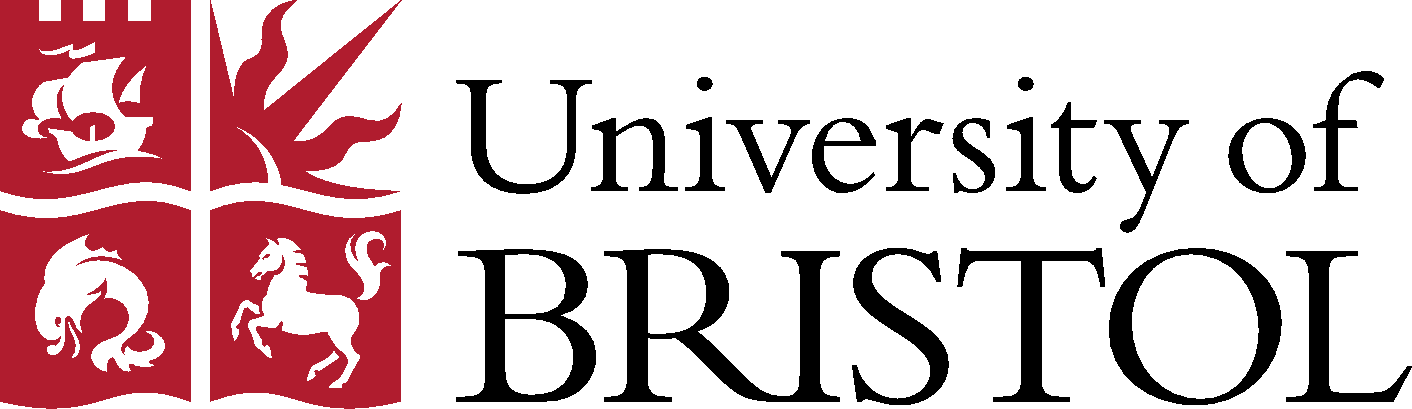 |
| --- | --- | --- |
|  | |  |

**Participant Information Sheet**

**HOPE:**

**Help for peOPle with money, Employment or benefit problems**

We are a group of researchers from Bristol University and we would like to invite you to take part in a research study that is evaluating a new HOPE Worker Service. Before you decide whether or not to take part, please take time to read the following information carefully and discuss it with your care team if you wish. This will help you understand why the research is being done and what it would involve for you.

**What is the purpose of the study?**

We would like to find out how best to help people who are struggling or experiencing distress due to financial hardship, debt, job loss or benefits worries. At the moment people in your situation are usually only given leaflets about support organisations when they leave hospital. We aim to see whether being given extra help to get relevant advice and support on these matters makes a difference to peoples’ wellbeing and financial situation.

**We want to compare two different ways of offering this support. The purpose of HOPE is to find out which of these methods of delivering a service is better by comparing the financial situation, mental health and wellbeing and the cost of each method.**

To do this you will be offered the support of a guide (or HOPE worker) who will talk with you about your situation and financial problems. Together you will decide on the best action to take and then will help you to get the support you need. This may be free debt advice, working out what benefits you are entitled to or helping you with any unopened letters or letters you find difficult to understand that you have received about your benefits or debts. The HOPE worker can also help you get an appointment to see your GP for IAPT (psychological therapy) if necessary. You will receive:

**Standard Service**

You and the HOPE worker will meet for one session of an hour and a half. OR

**Enhanced Service**

You and the HOPE worker will meet for up to 6 sessions of approximately an hour or so each

The aim is to have assisted you to move out of the crisis that caused your self-harm and hospital admission. The aim is also to help you to be more confident about dealing with any financial difficulties that may happen in the future.

**Who is organising and funding the research?**

The research is being organised locally by researchers based at the University of Bristol and staff employed by Avon and Wiltshire Mental Health Partnership NHS Trust (AWP) and University Hospitals NHS Foundation Trust (UHB) at the Bristol Royal Infirmary. The principal investigator, based at the University of Bristol, is Professor David Gunnell. The researcher you will have contact with is Maria Barnes. The research is funded by the NHS through the National Institute of Health Research and has been reviewed and approved by the Central Bristol Research Ethics Committee.

**Why have I been invited?**

You have been asked because of your recent hospital admission and contact with liaison psychiatry services at Bristol Royal Infirmary. We plan to offer the HOPE worker service to about 20 people for this study.

**Do I have to take part?**

You do not have to take part. Your care will not be affected by whatever you decide. We advise you to read this information carefully before you decide.

If you do decide to take part in the research a member of the psychiatric liaison team at the hospital will ask you to sign a consent form when you meet her, to show that we have your agreement to take part. However, you are free to change your mind at any time without needing to give a reason.

**What will happen if I take part?**

In this study, we need to compare people who have had 6 sessions with a HOPE worker with those who have had 1 session. We have summarised each of these services in the chart below and explained how you would be allocated to the study:

**Hospital**

A member of the liaison team at the hospital believes you may be someone who could benefit from the pilot service, so they give you an information sheet about the project.

If you are happy to take part, you sign a consent form agreeing to be phoned by the HOPE worker within the next 3 working days.

Stage 1

**Phone**

An appointment is made over the phone for the researcher and HOPE worker to meet you at home – or a place of your choice - as soon as possible

Stage 2

(within 3 days)

**Meeting with HOPE worker and Researcher**

The researcher and HOPE worker visit you to discuss the study in more depth.

- Randomisation is explained and you sign a second consent form agreeing to be randomly assigned to one of the services.
- A ‘baseline’ questionnaire is filled in.
- You will be randomised to the standard or enhanced HOPE worker service (this is done by computer)
- The researcher leaves and the session begins:

Stage 3

(within 7 days)

Stage 4

**Enhanced HOPE worker Service**

HOPE worker assesses with you what support services needed. This could include: debt advice, benefits entitlement, GP, psychological services, form filling, phone calls.

A plan and contract is made between the HOPE worker and participant for up to 6 sessions.

The HOPE worker keeps records of contact with participant, including advice given, contact with other organisations and actions agreed.

Sessions end after no more than 6 appointments.

**Standard HOPE worker Service**

HOPE worker assesses with you in one-off appointment what support services needed. This could include: debt advice, benefits entitlement, GP or, psychological services. The HOPE worker points you to relevant organisations and passes on written information.

The HOPE worker keeps a record of advice given to participant.

One-off session ends

**Stage 5 (after 3 months)**

**Follow-Up**

3 months after the first session researcher contacts participant

Questionnaire is completed again

Researcher interviews you about experience of HOPE worker service

**Service allocation**

The best way to compare these two services is to have similar groups of people allocated to either 6 sessions or 1 session, in a research study. The only way to make sure the groups of patients are as similar as possible is to have the allocation decided by chance: a process called randomisation. This means that you will have an equal chance of having either one session or six. This process ensures that the services are compared fairly. It is important for the study that you only agree to take part if you believe you would be prepared to accept either service. Whichever service you are allocated to, you will be treated with the best possible care and the HOPE worker will be very experienced.

**Recording Sessions**

One or more of your sessions may be recorded by the Hope worker. This is for training purposes only and makes sure that HOPE workers can improve their approach to working with people.

**What are the possible disadvantages of taking part?**

You may be allocated to the service getting one session and feel you require more. Or you may be allocated to six sessions and feel that this has taken up too much of your time. If you agree to take part, we will ask you to complete questionnaires and answer questions in an audio-recorded interview.

Talking about you feel about the service and what has happened might be upsetting or difficult for you. If this happens you can take a break or stop the appointment or interview at any time without giving a reason. You can also choose not to answer a question if you feel it is too personal or upsetting.

**What are the possible benefits of taking part?**

Whether you receive one session or up to six sessions, you will be receiving more support than is normally available for people who have self-harmed due to financial worries. You will be helping provide evidence for developing better support for people in similar situations to you in the future.

**Will my taking part in this study be kept confidential?**

What you tell us will be kept confidential to a small group of researchers working on this study. We will not use your name or identify you when we write about this study. With your permission we would like to let your care co-ordinator (which is usually your Community Psychiatric Nurse, Social Worker or GP) know that you are taking part in the study. We will be asking for consent to let this person know for their records only.

However, unless you say something which indicates that you or others are at significant risk of harm, we will not tell your care co-ordinator anything that you have said. We will copy you in on any correspondence we have with them regarding your participation in the study. If you have any concerns about confidentiality, please contact Maria (details below)

**Storage of Information**

Audio-recordings and typed copies of research interviews (“transcripts”) need to be kept for the duration of the project. Your name will not be attached to recordings or transcripts. All information will be kept securely in a locked filing cabinet for no longer than 15 years. We may use a direct quote from what you have said when reporting the results of the study but will make sure that no one is able to identify you and your name will not be shown.

**What will happen to the results of the research study?**

While the study is in progress, its early findings (and any other new relevant information) will be regularly monitored to ensure that the study remains safe and viable. The results will not be known for several months as we will need to analyse them carefully. The final results will be freely available on the study website and published in a scientific medical journal, but neither you nor other patients will be identified when this happens.

**What if there is a problem?**

- We aim to run this study with every care for everyone who takes part. However, if you have any concerns you have a number of choices.
  - We hope you feel you can talk directly to Maria on: 0117 3313929/mobile 07772 943256 or email: maria.barnes@bristol.ac.uk and ask her any questions about what we are doing. You can also talk to David Gunnell the lead researcher on the study on: 0117 9287253 or email: D.J.Gunnell@bristol.ac.uk.
  - If you are unhappy about what we have done, you can complain formally through the NHS complaints procedure. For further details contact: Avon and Wiltshire Partnership Mental Health Trust Complaints Department, Jenner House, Langley Park, Chippenham SN15 1GG. Tel: 01249 468261 or Freephone (from landline): 0800 073 1778
  - Although it is extremely unlikely, if you are harmed during the research due to someone’s negligence, then you may have grounds for a legal action for compensation against the University of Bristol but you may have to pay your legal costs.

**Thank you for taking the time to read this information. Please contact us if you have any questions or anything else you would like to discuss about the research.**
