# Supplementary material for: HOPE: Help fOr People with money, employment, benefit or housing problems: study protocol for a randomised controlled trial
Source: Pilot Feasibility Stud. 2017 Sep 19;3:44. doi: 10.1186/s40814-017-0179-y (PMC5629806; doi:10.1186/s40814-017-0179-y)
Supplement: Supplementary file 3 — Consent to contact. (DOCX 77 kb) [file 40814_2017_179_MOESM3_ESM.docx]

Identification Number:

**PERMISSION TO CONTACT FORM (1)**

**HOPE:**

**Help for peOPle with money, Employment or benefit problems**

*Please initial box*

Interviewee researcher

1. I have read and understood the information sheet for the above study

dated 29/02/2016 (Version 2).

2. I have had the opportunity to ask questions

3. Any questions I asked were answered satisfactorily

4. I understand that my participation is voluntary

5. I am free to withdraw at any time without giving a reason and

I do not have to answer questions I don’t want to

6. If I decide to withdraw from the study my medical care or

legal rights will not be affected.

7. I agree to contact with the researcher

8. I agree to contact with the HOPE worker

9. I consent to audio-recording of this consultation being kept

for staff training purposes only, and only when anonymised

10. I give permission for hospital staff to share risk information

with the service providers

Name of participant Date Signature

_______________________ _______________ __________________

Name of person taking consent Date Signature

Identification Number:
