# Supplementary material for: HOPE: Help fOr People with money, employment, benefit or housing problems: study protocol for a randomised controlled trial
Source: Pilot Feasibility Stud. 2017 Sep 19;3:44. doi: 10.1186/s40814-017-0179-y (PMC5629806; doi:10.1186/s40814-017-0179-y)
Supplement: Supplementary file 4 — Consent. (DOCX 76 kb) [file 40814_2017_179_MOESM4_ESM.docx]

Identification Number:

**CONSENT FORM (2)**

**HOPE:**

**Help for peOPle with money, Employment or benefit problems**

*Please initial box*

Interviewee researcher

1. I agree to be randomised to either of the arms of the study/service
2. I agree to the use of audio recording during the sessions and

the end interview

1. I agree to future contact by the researcher in the study
2. I would like a copy of the research findings *(optional)*
3. I agree to the research team having access to the HOPE worker

and medical records

1. I understand that relevant sections of my interview data

collected during the study, may be looked at by individuals

from the University of Bristol, from research regulatory authorities

or from the NHS Trust, where it is relevant to my taking part in

this research. I give permission for these individuals to have

access to my interview data.

1. I understand that the information collected about me will be used

to support other research in the future, and may be shared

Anonymously with other researchers

1. I understand that the interview is confidential and my name will

be anonymized in any written report about the study

Name of participant Date Signature

_________________________ _______________ __________________

Name of person taking consent Date Signature
